# Diurnal periodicity of conidia of aquatic hyphomycetes in water and entrapment on latex-coated slides in two South Indian streams

**DOI:** 10.1080/21501203.2016.1196759

**Published:** 2016-06-20

**Authors:** Sudeep D. Ghate, Kandikere R. Sridhar

**Affiliations:** Department of Biosciences, Mangalore University, Mangalore, India

**Keywords:** Aquatic hyphomycetes, diurnal periodicity, diversity, conidia, entrapment, latex, methods

## Abstract

Aquatic hyphomycete conidial trapping efficiency by the banyan (*F. benghalensis* L.) latex-coated glass slides was tested diurnally (3 h intervals) in the Western Ghats (Sampaje) and west coast (Konaje) streams in relation to abiotic factors (humidity, air temperature and water temperature). The conidial trapping efficiency of latex-coated slides was compared with plain glass slides and drift conidia in water. Three methods of assessment showed higher species richness, conidial richness and diversity in Sampaje than in Konaje stream. In both streams, species richness, conidial richness and diversity in latex-coated slides were the highest followed by conidia in water and plain slides. Three-way ANOVA revealed significant differences in overall species and conidial richness between the streams, sampling methods and time of sampling (*p *< 0.001). Multiple comparisons by Holm–Sidak test revealed significant differences in overall species and conidial richness between Sampaje and Konaje (*p *< 0.001); latex-coated slides and plain slides (*p *< 0.001); latex-coated slides and water filtration (*p *< 0.001); plain slides and water filtration (*p *< 0.001). Total species, total conidia and diversity assessed by the three methods peaked during 12 am–3 am in Sampaje stream, while during 3 am–6 am in Konaje stream. Cooler conditions due to relatively low water temperature favoured higher diversity of aquatic hyphomycetes in Sampaje than in Konaje stream. The three methods employed in the present study were not biased towards scolecoid or stauroid conidia. The top five species in both streams was composed of both types of conidia corroborating earlier annual or biannual studies in Konaje and Sampaje streams. Thus, assessment of population of aquatic hyphomycetes using banyan latex-coated slides will be advantageous over plain slides and drift conidia in streams.

## 1. Introduction

Aquatic hyphomycetes are geographically widespread mycota involved in detritus decomposition and mediates energy flow in streams (Bärlocher and Kendrick 1974; Suberkropp and Klug 1976; Gessner and Chauvet 1994; Duarte et al. 2016). One of the major adaptations of these fungi is production of morphologically distinct conidia (mainly scolecoid and stauroid) to drift, disseminate and anchor on to solid surfaces in streams (Webster and Davey 1984; Webster 1987; Dang et al. 2007; Kearns and Bärlocher 2008; Sridhar 2009). Different techniques are necessary to understand the assemblage, diversity and function of aquatic hyphomycetes in streams (e.g. conventional, biochemical and molecular methods). Evaluation of conidia in water, foam and detritus in streams will provide a fair idea on the assemblage and diversity of aquatic hyphomycetes.

Several microscopic, biochemical and molecular techniques are followed to study aquatic hyphomycetes in streams (Descals 1997; Graça et al. 2005; Bärlocher 2016). Some of the important conventional methods employed to study them in streams include: (i) Enumeration of drift conidia in water (microscopic observation and membrane filtration) (Iqbal and Webster 1973a; Müller-Haeckel and Marvanová 1979; Sridhar and Sudheep 2010); (ii) Conidial assessment in foam or scum (microscopic observation) (Ingold 1975); (iii) Conidial adhesion on artificial or natural traps (e.g. cellophane, plexiglass and rosin-coated slides and latex-coated slides) and microscopic assessment (Lindsey and Glover 1976; Müller-Haeckel and Marvanová 1976; Bärlocher et al. 1977; Ghate and Sridhar 2015); (iv) Direct microscopic examination of fungal growth and sporulation in substrata (incubation of segments of leaf detritus in distilled water in Petri plates) (Ingold 1942). Rosin-coated or latex-coated slides can also be employed as methods of choice as they efficiently trap conidia of aquatic hyphomycetes in streams (Bärlocher et al. 1977; Ghate and Sridhar 2015).

Activities of aquatic hyphomycetes in streams are strongly dependent on seasonal input of leaf litter into streams (Bärlocher 1992). However, such seasonal fluctuations vary depending on geographic locations (Sridhar and Kaveriappa 1984, 1989a; Bärlocher 1992, 2000). In southwestern India, major spell of aquatic hyphomycetes occur during monsoon and post-monsoon periods (Sridhar et al. 1992). Although dynamics of aquatic hyphomycetes have been investigated based on drift conidia in different parts of the world (Müller-Haeckel and Marvanová 1979; Shearer and Lane 1983; Aimer and Segedin 1985; Shearer and Webster 1985; Thomas et al. 1989; Fabre 1998; Gönczöl and Révay 1999; Gönczöl et al. 2001), studies on diurnal fluctuations of drift conidia in streams seem to be scanty (Thomas et al. 1991, 1992; Sridhar and Sudheep 2010; Ghate and Sridhar 2015). Among the six latexes tested, latex derived from banyan tree species (*Ficus benghalensis*) proved excellent in adhesion of conidia (Ghate and Sridhar 2015). In this follow-up study we compared the pattern of diurnal fluctuation of conidia of aquatic hyphomycetes by using three methodologies: (i) under drift in water, (ii) adhesion on plain glass slides and (iii) adhesion on banyan latex-coated glass slides in typical coastal and Western Ghat streams. We hypothesise that trapping conidia of aquatic hyphomycetes using latex-coated slides will be superior over other methods followed in this study.

## 2. Materials and methods

### 2.1. Streams

The experiments for assessing aquatic hyphomycetes drift conidia, conidial adhesion on plain glass slides and conidial adhesion on banyan latex-coated glass slides were carried out during post-monsoon period (Konaje: 3–4 October 2014; Sampaje: 11–12 October 2014). The streams chosen were: (i) a second-order seasonal coastal stream flowing through the laterite terrain and mixed plantations adjacent to the Mangalore University Campus, the Konaje (12°48′N, 74°55′E; 90 m asl) and (ii) a third-order perennial stream flowing through mixed forests with partially rocky terrain in the Western Ghats, the Sampaje (12°29′N, 75°35′E; 500 m asl). Samples were assessed at five sites in the two streams at a distance of about 25 m.

### 2.2. Abiotic factors

At each stream site, miniscule temperature and humidity were assessed using a digital thermohygrometer (Mextech Digital Thermohygrometer, Mumbai). Water temperature was measured by using a glass mercury thermometer (N.S. Dimple Thermometer, New Delhi).

### 2.3. Conidial drift and adhesion

Aliquots of water samples (125 ml in 25 ml aliquots) during each interval of diurnal study were filtered (Millipore filters: porosity 5 μm; diam., 25 mm) and fixed with 1% lactophenol aniline blue. Filters were later mounted on slides with lactic acid and scanned using a light microscope (Nikon YS100, Nikon Corporation, Tokyo) for assessment of the identity of the conidia of aquatic hyphomycetes by using taxonomic monographs (Ingold 1975; Nawawi 1985; Marvanová 1997; Santos-flores and Betancourt-Lopez 1997).

Latex was collected by cutting petioles of mature green leaves of *Ficus benghalensis* L. (Moraceae), one or two drops of latex oozing from petiole was collected on sterile glass slide and smeared with another sterile slide similar to blood smear preparation. The smears were allowed to air-dry at laboratory temperature (27.5–29.5°C) up to 12 h before immersing in stream.

### 2.4. Diurnal periodicity

Experiments were started at 9 am with water filtration and immersion of slides. Latex-coated slides were vertically mounted side by side in grooves of thermocol sheet and placed in perforated plastic boxes. The boxes were immersed by tying to tree trunks/roots and their position was set in stream to expose latex-coat to face flowing water. Simultaneously another set of plastic boxes containing five plain slides were also introduced as control slides. Thereafter, at every 3 h interval up to 24 h (9 am) the same procedure was repeated followed by retrieval of the previously immersed slides. An additional trial was carried out beyond 24 h (i.e. at 12 pm).

At every 3 h intervals, slides were retrieved from stream, stained with aniline blue (0.1%) in lactophenol by applying cover glass (40 × 22 mm) and preserved in slide boxes. The slides were observed for the conidia of aquatic hyphomycetes adhered below the cover glass area (880 mm^2^) by microscopic examination. The conidia were identified based on the conidial morphology as described earlier.

### 2.5. Data analysis

Percentage contribution of each species was calculated for drift conidia in water, adhered conidia on plain slides and adhered conidia on latex-coated slides. The diversity of aquatic hyphomycete conidia in drift and adhered to slides was evaluated by Shannon’s diversity (Magurran 1988) and Pielou’s equitability (Pielou 1975). To assess similarity of aquatic hyphomycete communities using the three sampling methods in Konaje and Sampaje streams, Sørensen’s similarities (%) were calculated (Chao et al. 2005). Three-way ANOVA was employed to assess the impact of the streams (Konaje and Sampaje), the sampling methods employed (water filtration, adhesion on plain slides and adhesion on latex-coated slides) and time of sampling (diurnal pattern) on aquatic hyphomycete species and conidial richness (SigmaPlot, version # 11, Systat Inc., USA). Multiple comparisons were performed using Holm–Sidak post-tests.

## 3. Results

### 3.1. Abiotic features

**Figure 1.**
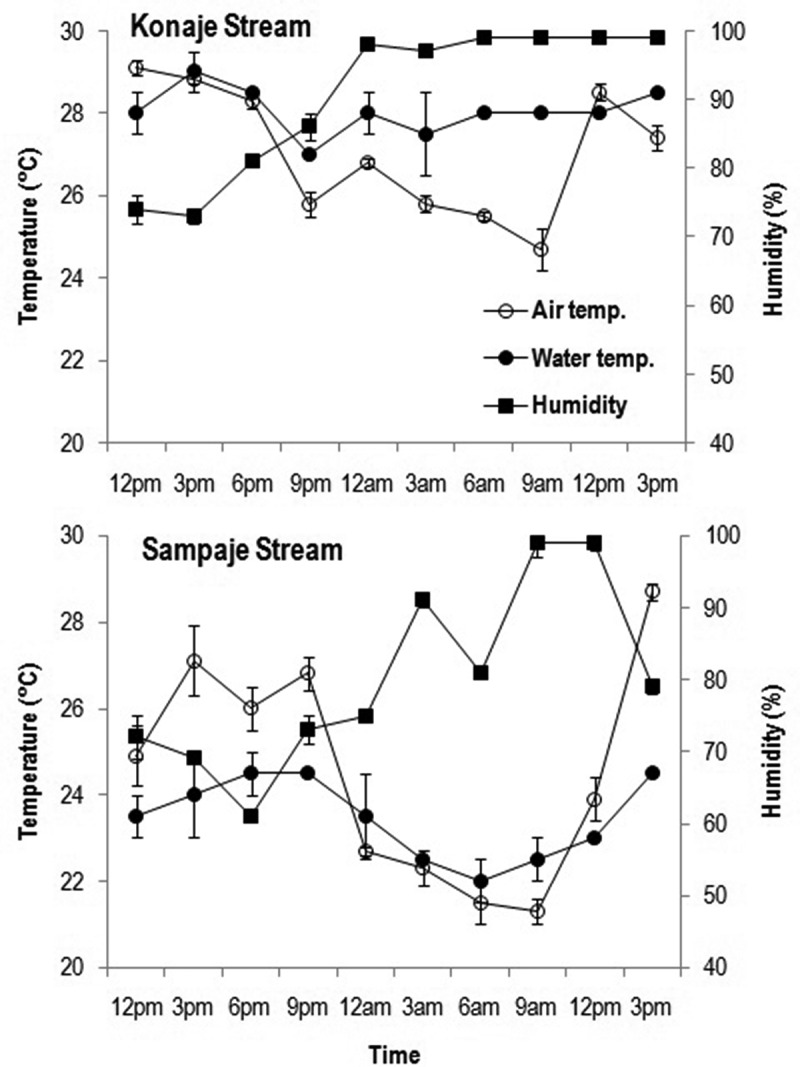
Fluctuation in air temperature, water temperature and humidity during diurnal study of aquatic hyphomycetes in Konaje and Sampaje streams.

**Figure 2.**
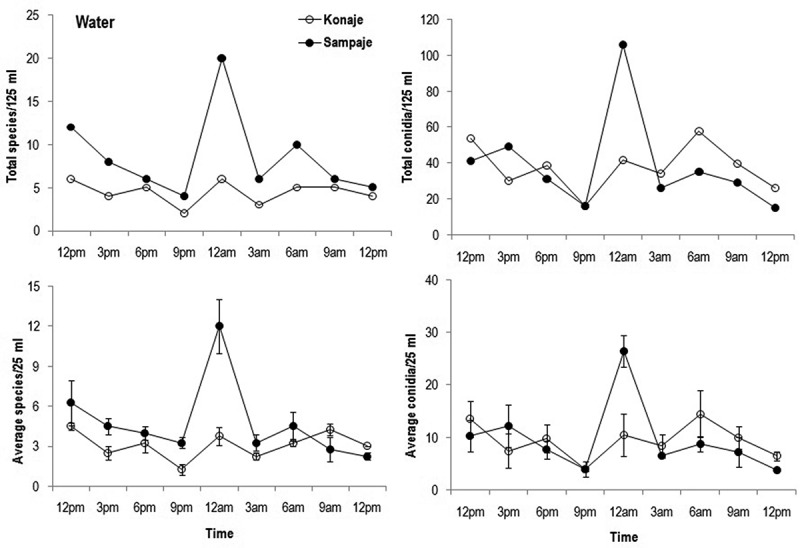
Fluctuation in total and average number of species; total and average number of conidia in water during diurnal study of aquatic hyphomycetes in Konaje and Sampaje streams.

In Konaje stream, the air temperature was highest at 12 pm (29.1°C) and thereafter tended to gradually decrease (Figure 1). The humidity was lowest at 3 pm (73%), gradually increased till saturation at 12 am and thereafter (97–99%). The water temperature was lowest at 9 pm (27°C) and highest at 3 pm (29°C). In Sampaje stream, the air temperature was highest at 3 pm (27.1–28.7°C) and lowest at 9 am (21.3°C). The humidity was lowest at 6 pm (61%) and attained saturation at 9 am and 12 pm (99%). The water temperature was lowest at 6 am (22°C) and highest at 6 pm and 9 pm (24.5°C) (Figure 2).

### 3.2. Conidial drift

Total species (4–20 vs. 2–8 spp.) as well as average species (2.3–12 vs. 1.3–4.5 spp.) in water were higher in Sampaje than Konaje stream (Figure 2). Fluctuation of the number of species as well as average number of species were also clear cut in Sampaje compared to Konaje stream. Total (16–58 vs. 15–106) and average (4–14.5 vs. 4–26.5) number of conidia were higher in Sampaje than in Konaje stream. Total and average number of conidia peaked at 6 am in Konaje stream, while during 12 am in Sampaje stream. Top five species contributing for total conidia found in water of Konaje stream in decreasing order were *Lunulospora curvula, Flagellospora curvula, Anguillospora longissima, Triscelophorus monosporus* and *Flagellospora penicillioides*, while in Sampaje stream were: *Lunulospora cymbiformis, F. curuvla, A. longissima, Clavatospora tentacula* and *F. penicillioides* (Table S1). Conidia of *Lunulospora curvula* and *L. cymbiformis* were represented in all sampling time of Konaje and Sampaje streams, respectively.

### 3.3. Conidial adhesion on plain slides

**Figure 3.**
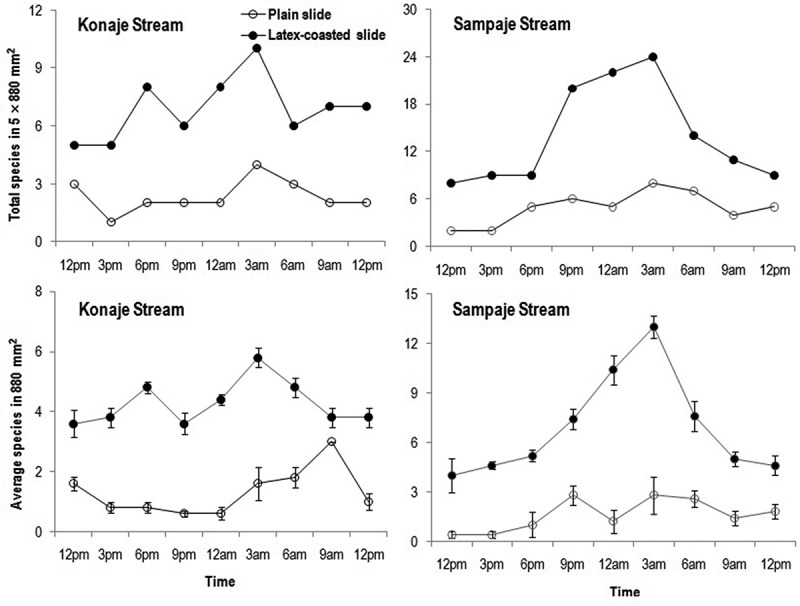
Fluctuation in total and average number of species adhered on plain glass slides and latex-coated slides during diurnal study of aquatic hyphomycetes in Konaje and Sampaje streams.

**Figure 4.**
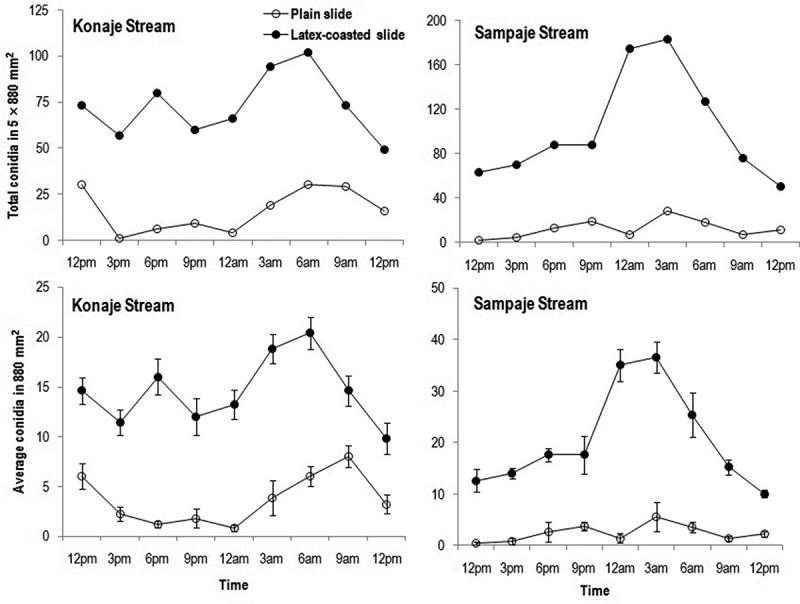
Fluctuation in total and average number of conidia adhered on plain glass slides and latex-coated slides during diurnal study of aquatic hyphomycetes in Konaje and Sampaje streams.

The total number of species (2–8 vs. 1–4 spp.) that adhered to plain slides were higher in Sampaje than in Konaje stream, while the opposite was found for average species number (0.4–2.8 vs. 0.6–3 spp.) (Figure 3). Total and average number of species in Konaje stream peaked at 3 am and 9 am, respectively, while in Sampaje stream it was at 3 am. Adherence of conidia in Sampaje and Konaje streams was almost similar (2–28 vs. 1–30), but the average number of conidia was lower in Sampaje than in Konaje stream (0.4–5.6 vs. 0.8–8) (Figure 4), for plain slides. Total and average conidia in Konaje stream peaked at 12 pm and 6 am/9 am. Sampaje stream showed two peaks in total and average conidia during 9 pm and 3 am. Top five species adhered on plain glass slides in Konaje stream in decreasing order were: *Lunulospora curvula, Triscelophorus monosporus, Phalangispora constricta, Flagellospora curvula* and *Trifurcospora irregularis*, while in Sampaje stream were: *Lunulospora cymbiformis, Anguillospora longissima, Triscelophorus acuminatus, F. curuvla* and *L. curvula* (Table S2).

### 3.4. Conidial adhesion on latex

The total number of species (8–24 vs. 5–10 spp.) and average number of species (3.6–5.8 vs. 4–13 spp.) adhered to latex-coated slides were higher in Sampaje than Konaje stream (Figure 3). The total as well as the average number of species showed two peaks at 6 pm and 3 am in Konaje stream, while in Sampaje showed only one peak at 3 am. Adhesion of total number of conidia (50–183 vs. 49–102) and average number of conidia (10–36.6 vs. 9.8–20.4) were higher in Sampaje than in Konaje stream (Figure 4). Total and average number of conidia peaked at 6 pm and 6 am in Konaje stream, while in Sampaje the highest number of conidia was at 3 am. The top five species adhered on latex-coated slides in Konaje stream in decreasing order were *Lunulospora curvula, Triscelophorus monosporus, Flagellospora curvula, Anguillospora longissima* and *Phalangispora constricta*, while in Sampaje stream were: *Triscelophorus acuminatus, Lunulospora cymbiformis, A. longissima, L. curvula* and *F. curvula* (Table S3). *Anguillospora longissima* was represented in all sampling times in Konaje as well as in Sampaje streams and in addition, *Triscelophorus acuminatus* was also found in all sampling times in Sampaje stream.

### 3.5. Diversity, similarity and cumulative species

**Figure 5.**
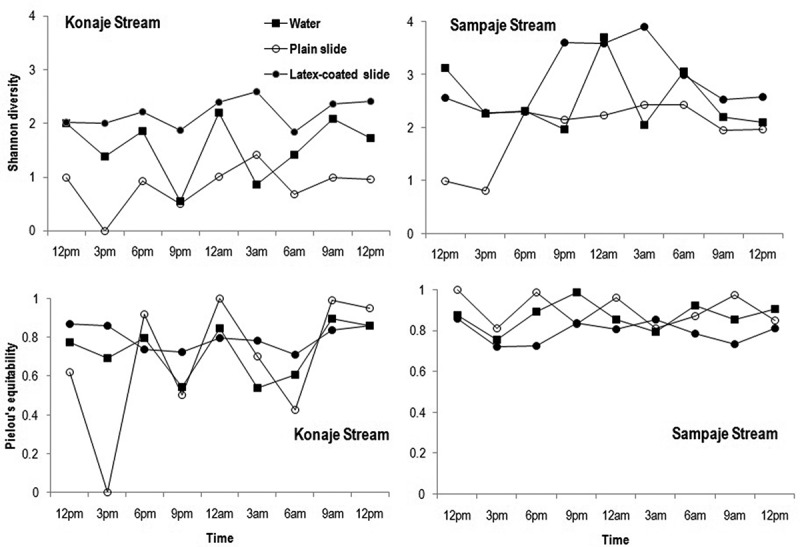
Shannon diversity and Pielou’s equitability of aquatic hyphomycetes in water, plain glass slides and latex-coated slides during diurnal study in Konaje and Sampaje streams.

Shannon diversity in latex-coated slides was consistently higher compared to water filtration and adherence on plain slides with a major peak at 3 am on Konaje as well as Sampaje stream (Figure 5). The diversity was lowest based on adhesion to plain slides in both streams. Pielou’s equitability was inconsistent especially in plain slides in Konaje stream, while it remained more or less uniform in Sampaje stream.

Sørensen’s similarity was higher between aquatic hyphomycete communities assessed by plain slides and latex-coated slides (69.6%) in Konaje stream, as well as in Sampaje stream (80.7%) compared with communities on latex-coated slides and found through water filtration (58.4% and 74.5%, respectively).

**Figure 6.**
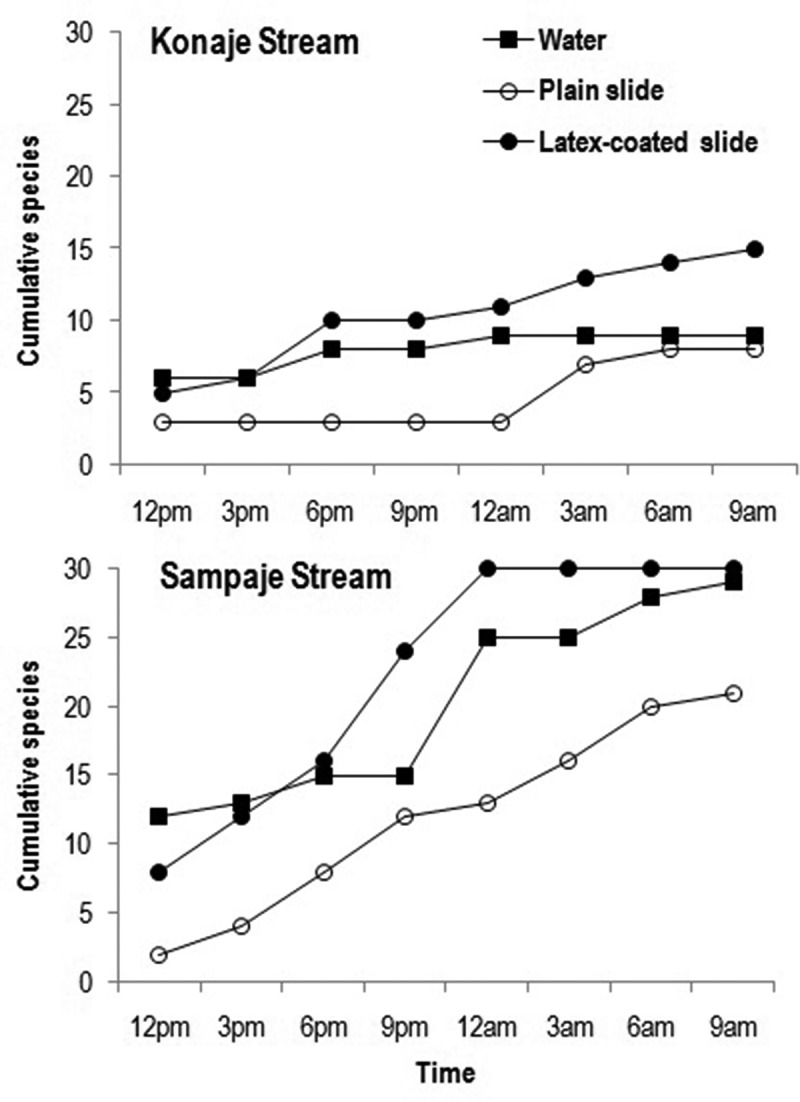
Cumulative species of number of aquatic hyphomycetes in water, plain glass slides and latex-coated slides during diurnal study in Konaje and Sampaje streams.

The cumulative number of species assessed by the three methods in Konaje and Sampaje streams at 3 h interval time points revealed that latex-coated slides presented the higher number of species than the one found through water filtration or adhesion to plain slides (Figure 6).

### 3.6. Comparison of streams and methods

Overall species and conidial richness were higher in Sampaje than in Konaje stream (Tables S1–S3). By using any of the three methods for diversity assessment, the richness of species and conidia were also consistently high in Sampaje stream (Figures 2–4). Among the three sampling methods, latex-coated slides had the highest richness of species adhered as well as conidia, followed by water filtration and plain glass slides.

Three-way ANOVAs revealed significant differences in overall species and conidial richness between the streams, sampling methods and time (Three-way ANOVA, *p *< 0.001) (Table 1). Holm–Sidak multiple comparison tests revealed significant differences in overall species and conidial richness between Sampaje and Konaje; plain slides and latex-coated slides; plain slides and water filtration; latex-coated slides and water filtration (Holm–Sidak post-tests, *p *< 0.001).

**Table 1.** Three-way ANOVA of the impact of stream (Konaje and Sampaje), method (water filtration, plain slides and latex-coated slides) and time on richness of species and conidia of aquatic hyphomycetes (df, degrees of freedom; F, ratio of two mean square values; *p*, level of significance).

Treatment	Species richness			Conidial richness		
	df	F	*p*	df	F	*p*
Stream	1	89.994	<0.001	1	11.459	<0.001
Method	2	220.352	<0.001	2	255.205	<0.001
Time	8	14.426	<0.001	8	14.746	<0.001
Stream × Method	2	16.006	<0.001	2	17.822	<0.001
Stream × Time	8	12.147	<0.001	8	10.024	<0.001
Sample × Time	16	10.438	<0.001	16	7.988	<0.001
Stream × Method × Time	16	4.193	<0.001	16	2.892	<0.001

Among the peak periods of species and conidial richness (12 am, 3 am and 6 am), a significant difference was seen between 12 am vs. 12 pm (27 h), 3 pm, 6 pm, 9 pm and 9 am; 3 am vs. 12 pm (27 h), 3 pm, 9 am, 6 pm, 9 pm and 12 pm; 6 am vs. 12 pm (27 h) and 3 pm (Holm–Sidak post-tests, *p *< 0.001). Significant difference in conidial richness between 6 am vs. 9 pm, 9 am, 6 pm and 12 pm (Holm–Sidak post-tests, *p *< 0.001) was also found.

At 12 am, there was a significant difference in species richness between Sampaje and Konaje; water filtration and plain slides; latex-coated slides and plain slides (Holm–Sidak post-tests, *p *< 0.001). At 3 am, there was a significant difference in species and conidial richness between Sampaje and Konaje; latex-coated slides and water filtration; latex-coated slides and plain slides (Holm–Sidak post-tests, *p *< 0.001). At 6 am, there was a significant difference in species richness between latex-coated slides and water filtration (Holm–Sidak post-tests, *p *< 0.001); latex-coated slides and plain slides; water filtration and plain slides (Holm–Sidak post-tests, *p *< 0.01).

At 12 am, there was a significant difference in conidial richness between Sampaje and Konaje stream; water filtration and plain slides; latex-coated slides and plain slides in Konaje; water filtration and plain slides; latex-coated slides and plain slides; latex-coated slides and water filtration in Sampaje (Holm–Sidak post-tests, *p *< 0.001). At 6 am, there was a significant difference in conidial richness between Sampaje and Konaje; latex-coated slides and water filtration; latex-coated slides and plain slides; water filtration and plain slides (Holm–Sidak post-tests, *p *< 0.001).

## 4. Discussion

Temporal studies on aquatic hyphomycetes in streams ranges from diurnal to half a decade (e.g. diurnal: Thomas et al. 1991; 1992; Sridhar and Sudheep 2010; annual: Sridhar and Kaveriappa 1992; biannual: Sridhar and Kaveriappa 1984, 1989a, 1989b; 5 years: Bärlocher 2000). Similarly, a wide range of techniques are employed to assess aquatic hyphomycetes in streams (microscopic, biochemical and molecular methods) (see Graça et al. 2005). Although sophisticated techniques are followed, simple methods are still valid for exploratory surveys in streams of different geographic locations (e.g. water filtration, foam observation and leaf litter observation) (Descals 1997). One of the basic requirements to follow morphological diversity of aquatic hyphomycetes and taxonomic descriptions is microscopic observations (conidial morphology and ontogeny) (Ingold 1975). Studies on conidial morphology and ontogeny resulted in description of diverse aquatic hyphomycetes throughout the world (Ingold 1975; Marvanová 1997; Gulis et al. 2005). Considering adhesion property of conidia, a variety of techniques have been designed for assessment of aquatic hyphomycetes (e.g. cellophane, collodion-coated glass rods, latex-coated slides, plexiglass and rosin-coated slides) (Webster 1959; Lindsey and Glover 1976; Müller-Haeckel and Marvanová 1976; Bärlocher et al. 1977; Ghate and Sridhar 2015).

Although diurnal studies span for a short duration (24 h), it will provide a fair idea of aquatic hyphomycete community in streams. For instance, mere water filtration up to 24 h in Konaje stream resulted in identification of 19 species (Sridhar and Sudheep 2010), which is comparable to long-term annual studies using more than one technique (Sridhar and Kaveriappa 1984, 1989a). Thus, diurnal study serves as a quick approach to assess richness and diversity of aquatic hyphomycetes in streams. In the present study, 50% of species reported by biannual studies in Konaje stream were represented in water filtration and immersion of plain slides, while in latex-coated slides it was up to 83% of reported species. Interestingly, in an earlier diurnal study in Konaje stream by water filtration, 100% of aquatic hyphomycetes were recovered (Sridhar and Sudheep 2010). In Sampaje stream, adhesion on plain slides revealed 29% of species found in annual studies, 38% and 40% in water filtration and latex-coated slides, respectively. Cumulative species at different sampling intervals showed that latex-coated slides possess a higher ability to adhere conidia than plain slides and diversity was even higher than found through the water filtration. The present study showed relatively poor conidial richness on plain slides, moderate in water filtration and highest in latex-coated slides. Thus, banyan latex-coated slides have a similar potential to assess the richness of aquatic hyphomycetes in water than the water filtration technique.

Conidial shape has been modified from conventional configuration (cylindroid/ovoid) into scolecoid (sigmoid/filiform) or stauroid (staurosporus/multiradiate) as a result of parallel and or convergent evolution (Ingold 1975). The ovoid conidia have least points of attachment (1) compared to scolecoid (2) and stauroid (at least 3) conidia (Webster 1959; Webster and Davey 1984). Increase in conidial length also increases the probability of attachment on solid surfaces (Cox 1983). There is a notion that buoyancy as well as trapping efficiency of stauroid conidia in air bubbles (in foam) is better than that found for ovoid and scolecoid conidia (Iqbal and Webster 1973b). On the contrary, observations made in this study as well as in an earlier study (Ghate and Sridhar 2015) showed that water filtration, adhesion on plain slides and latex-coated slides are not biased towards conidial shape (Sridhar and Sudheep 2010; Ghate and Sridhar 2015). In this study, top five species in all methods encompass scolecoid as well as stauroid conidia; moreover, they were also top in frequency of occurrence based on annual or biannual observations in Konaje and Sampaje streams (with an exception of *Trifurcospora irregularis* in Konaje stream). Thus, banyan latex-coated slides serve as unbiased trapping surface for conidia of aquatic hyhomycetes in streams.

The extent of diurnal fluctuation of abiotic factors (humidity, air temperature and water temperature) was narrower in Konaje compared to Sampaje due to altitudinal and geographic difference. Air temperature and humidity are opposing at night, same feature pertain to water temperature in Sampaje stream, but not in Konaje stream. The diurnal fluctuation of species and conidia of aquatic hyphomycetes in two coastal streams (Sridhar and Sudheep 2010) almost corroborated with the present study. Overall, species as well as conidia peaked during night than during day in the present as well as earlier study in Konaje stream (Sridhar and Sudheep 2010). We predict that significant increase of air temperature and water temperature might result in reduced species and conidial output due to induction of vegetative phase during day and reproductive phase during night. This is contradictory to the observations in temperate streams in Australia by the activity of leaf shredders (Thomas et al. 1991, 1992). Observations over three decades of Konaje and Sampaje streams revealed no leaf-shredding invertebrates. Increased number of species as well as conidia of aquatic hyphomycetes during night hours may be due to lack of invertebrate activities. Relatively, cooler conditions, especially low water temperature favours higher diversity of aquatic hyphomycetes in Sampaje than in Konaje stream. Besides, forest cover and the extent of organic matter input to Sampaje stream are higher than Konaje stream (Sridhar and Kaveriappa 1984, 1989b). Based on the present study and earlier temporal studies (Sridhar and Kaveriappa 1984; Sridhar and Kaveriappa 1989a, 1989b, 1992; Sridhar and Sudheep 2010; Ghate and Sridhar 2015), Sampaje stream possesses higher species richness, conidial richness and diversity than Konaje stream.

The present study supported the proposed hypothesis that assessment of aquatic hyphomycetes in streams using banyan latex-coated slides will be simple and efficient over other methods. Thus, conidial trapping by latex-coated slides could be a method of choice for evaluation of aquatic hyphomycete community in streams worldwide.
