# Supplementary material for: Diurnal periodicity of conidia of aquatic hyphomycetes in water and entrapment on latex-coated slides in two South Indian streams
Source: Mycology. 2016 Jun 20;7(2):88–97. doi: 10.1080/21501203.2016.1196759 (PMC6059061; doi:10.1080/21501203.2016.1196759)
Supplement: Supplementary_material.zip [file TMYC_A_1196759_SM3838.zip › Supplementary material/Table_S1.docx]

**Table S1.** Percent contribution of drift conidia of aquatic hyphomycetes in Konaje and Sampaje streams (n=5) (arranged in descending order).

|  | 9am | 12pm | 3pm | 6pm | 9pm | 12am | 3am | 6am | 9am | 12pm |
| --- | --- | --- | --- | --- | --- | --- | --- | --- | --- | --- |
| Konaje stream |  |  |  |  |  |  |  |  |  |  |
| *Lunulospora curvula* Ingold | 26.9 | 46.3 | 60.0 | 53.8 | 87.5 | 38.1 | 79.4 | 65.5 | 45.0 | 30.8 |
| *Flagellospora curvula* Ingold | 15.4 | 25.9 | 30.0 | 20.5 | − | 26.2 | − | 20.7 | 17.5 | 42.3 |
| *Anguillospora longissima* (Sacc. & P. Syd.) Ingold | 46.2 | 11.1 | 3.3 | 7.7 | − | 7.1 | − | 1.7 | 12.5 | 23.1 |
| *Triscelophorus monosporus* Ingold | 3.8 | 3.7 | − | − | 12.5 | 16.7 | 17.6 | 10.3 | − | − |
| *Flagellospora penicillioides* Ingold | 3.8 | 11.1 | − | 7.7 | − | 9.5 | 2.9 | − | − | − |
| *Cylindrocarpon* sp. | − | − | − | 10.3 | − | − | − | − | 12.5 | − |
| *Anguillospora crassa* Ingold | − | − | − | − | − | 2.4 | − | 1.7 | 12.5 | 3.8 |
| *Clavariopsis aquatica* De Wild. | − | 1.9 | 6.7 | − | − | − | − | − | − | − |
| *Dendrospora* sp. | 3.8 | − | − | − | − | − | − | − | − | − |
| Sampaje stream |  |  |  |  |  |  |  |  |  |  |
| *Lunulospora cymbiformis* K. Miura | 25.0 | 24.4 | 38.8 | 9.7 | 25.0 | 22.6 | 30.8 | 17.1 | 41.4 | 20.0 |
| *Flagellospora curvula* Ingold | 19.4 | 19.5 | 6.1 | 16.1 | − | 16.0 | 42.3 | 17.1 | 17.2 | 20.0 |
| *Anguillospora longissima* (Sacc. & P. Syd.) Ingold | 11.1 | 9.8 | 32.7 | 41.9 | 25.0 | 7.5 | − | 11.4 | 3.4 | 40.0 |
| *Clavatospora tentacula* Sv. Nilsson | − | 9.8 | 8.2 | 12.9 | − | 7.5 | − | 2.9 | − | − |
| *Flagellospora penicillioides* Ingold | 8.3 | − | − | 9.7 | 18.8 | − | 3.8 | − | 20.7 | 13.3 |
| *Campylospora chaetocladia* Ranzoni | 13.9 | 4.9 | 6.1 | − | − | 4.7 | − | − | 6.9 | − |
| *Anguillospora crassa* Ingold | − | 9.8 | 2.0 | − | 31.3 | 0.9 | 7.7 | − | − | − |
| *Lunulospora curvula* Ingold | − | 9.8 | − | − | − | 2.8 | 3.8 | 8.6 | − | − |
| *Clavariopsis aquatica* De Wild. | 5.6 | − | 2.0 | − | − | 0.9 | 11.5 | 5.7 | − | − |
| *Flabellospora crassa* Alas. | − | − | − | 9.7 | − | 5.7 | − | − | − | − |
| *Triscelophorus acuminatus* Nawawi | 8.3 | 2.4 | − | − | − | 4.7 | − | − | − | − |
| *Clavariopsis azlanii* Nawawi | − | − | − | − | − | − | − | 20.0 | − | − |
| *Heliscella stellata* (Ingold & V.J. Cox) Marvanová | 8.3 | − | − | − | − | − | − | − | 10.3 | 6.7 |
| *Condylospora spumigena* Nawawi | − | − | − | − | − | 3.8 | − | 5.7 | − | − |
| *Isthmotricladia laeensis* Matsush. | − | − | − | − | − | 5.7 | − | − | − | − |
| *Campylospora* sp. | − | − | − | − | − | 4.7 | − | − | − | − |
| *Brachiosphaera tropicalis* Nawawi | − | − | − | − | − | 3.8 | − | − | − | − |
| *Dendrosporium lobatum* Plakidas & Edgerton ex J.L. Crane | − | − | − | − | − | − | − | 8.6 | − | − |
| *Dwayaangam* sp. | − | 2.4 | 4.1 | − | − | − | − | − | − | − |
| *Triscelophorus konajensis* | − | − | − | − | − | 2.8 | − | − | − | − |
| *Trisulcosporium* sp. | − | − | − | − | − | 1.9 | − | − | − | − |
| *Campylospora parvula* Kuzuha | − | − | − | − | − | 0.9 | − | − | − | − |
| *Diplocladiella scalaroides* G. Arnaud ex M.B. Ellis | − | 2.4 | − | − | − | − | − | − | − | − |
| *Helicosporium* sp. | − | 2.4 | − | − | − | − | − | − | − | − |
| *Lemonniera terrestris* Tubaki | − | − | − | − | − | 0.9 | − | − | − | − |
| *Phalangispora bharathensis* T.S.K. Prasad & Bhat | − | − | − | − | − | 0.9 | − | − | − | − |
| *Tetracladium* sp. | − | − | − | − | − | 0.9 | − | − | − | − |
| *Triscelophorus monosporus* Ingold | − | 2.4 | − | − | − | − | − | − | − | − |
| *Trifurcospora irregularis* (Matsush.) K. Ando & Tubaki | − | − | − | − | − | − | − | 2.9 | − | − |
