# Supplementary material for: Diurnal periodicity of conidia of aquatic hyphomycetes in water and entrapment on latex-coated slides in two South Indian streams
Source: Mycology. 2016 Jun 20;7(2):88–97. doi: 10.1080/21501203.2016.1196759 (PMC6059061; doi:10.1080/21501203.2016.1196759)
Supplement: Supplementary_material.zip [file TMYC_A_1196759_SM3838.zip › Supplementary material/Table_S2.docx]

**Table S2.** Percent contribution of aquatic hyphomycetes in Konaje and Sampaje streams based on conidial adhesion on plain glass slides (n=5) (arranged in descending order).

.

|  | 12pm | 3pm | 6pm | 9pm | 12am | 3am | 6am | 9am | 12pm |
| --- | --- | --- | --- | --- | --- | --- | --- | --- | --- |
| Konaje stream |  |  |  |  |  |  |  |  |  |
| *Lunulospora curvula* Ingold | 73.3 | 100 | 66.7 | 88.9 | 50.0 | − | 86.7 | 44.8 | 62.5 |
| *Triscelophorus monosporus* Ingold | 23.3 | − | 33.3 | 11.1 | 50.0 | − | 10.0 | 55.2 | − |
| *Phalangispora constricta* Nawawi & J. Webster | − | − | − | − | − | 63.2 | − | − | − |
| *Flagellospora curvula* Ingold | − | − | − | − | − | 5.3 | − | − | 37.5 |
| *Trifurcospora irregularis* (Matsush.) K. Ando & Tubaki | − | − | − | − | − | 26.3 | − | − | − |
| *Clavariana aquatica* Nawawi | − | − | − | − | − | − | 3.3 | − | − |
| *Triscelophorus konajensis* K.R. Sridhar & Kaver. | 3.3 | − | − | − | − | − | − | − | − |
| *Ypsilina graminea* (Ingold, P.J. McDougall & Dann)  Descals, J. Webster & Marvanová | − | − | − | − | − | 5.3 | − | − | − |
| Sampaje stream |  |  |  |  |  |  |  |  |  |
| *Lunulospora cymbiformis* K. Miura | − | − | − | − | 28.6 | 36.4 | 38.9 | 28.6 | − |
| *Anguillospora longissima* (Sacc. & P. Syd.) Ingold | − | 75.0 | − | 15.8 | 28.6 | 15.2 | − | − | 27.3 |
| *Triscelophorus acuminatus* Nawawi | − | − | 23.1 | 31.6 | 14.3 | 12.1 | − | − | − |
| *Flagellospora curvula* Ingold | − | − | − | − | − | − | 16.7 | 28.6 | 45.5 |
| *Lunulospora curvula* Ingold | 50 | − | 23.1 | − | 14.3 | − | 11.1 | − | − |
| *Triscelophorus konajensis* | − | − | − | 36.8 | − | − | − | − | − |
| *Campylospora chaetocladia* Ranzoni | − | − | − | − | − | 6.1 | 16.7 | − | 9.1 |
| *Triscelophorus monosporus* Ingold | − | − | 15.4 | − | − | 6.1 | − | 28.6 | − |
| *Helicosporium* sp. | − | − | 23.1 | − | 14.3 | 3.0 | − | − | − |
| *Flabellospora crassa* Alas. | − | − | − | 5.3 | − | − | − | − | 9.1 |
| *Subulispora* sp. | − | − | 15.4 | − | − | − | − | − | − |
| *Anguillospora crassa* Ingold | − | − | − | − | − | − | − | 14.3 | − |
| *Brachiosphaera tropicalis* Nawawi | − | − | − | − | − | 3.0 | − | − | − |
| *Campylospora* sp. | − | − | − | − | − | − | 5.6 | − | − |
| *Clavatospora tentacula* Sv. Nilsson | − | − | − | 5.3 | − | − | − | − | − |
| *Condylospora spumigena* Nawawi | − | − | − | − | − | − | 5.6 | − | − |
| *Cylindrocarpon* sp. | − | 25.0 | − | − | − | − | − | − | − |
| *Flabellospora multiradiata* Nawawi | − | − | − | 5.3 | − | − | − | − | − |
| *Flabellospora verticillata* Alas. | 50.0 | − | − | − | − | − | − | − | − |
| *Magdalaenaea Monogramma* G. Arnaud | − | − | − | − | − | − | 5.6 | − | − |
| *Phalangispora constricta* Nawawi & J. Webster | − | − | − | − | − | 3.0 | − | − | − |
| *Trifurcospora irregularis* (Matsush.) K. Ando & Tubaki | − | − | − | − | − | − | − | − | 9.1 |
