# Supplementary material for: Diurnal periodicity of conidia of aquatic hyphomycetes in water and entrapment on latex-coated slides in two South Indian streams
Source: Mycology. 2016 Jun 20;7(2):88–97. doi: 10.1080/21501203.2016.1196759 (PMC6059061; doi:10.1080/21501203.2016.1196759)
Supplement: Supplementary_material.zip [file TMYC_A_1196759_SM3838.zip › Supplementary material/Table_S3.docx]

**Table S3.** Percent contribution of aquatic hyphomycetes in Konaje and Sampaje streams based on adhesion on latex-coated glass slides (n=5) (arranged in descending order).

|  | 12pm | 3pm | 6pm | 9pm | 12am | 3am | 6am | 9am | 12pm |
| --- | --- | --- | --- | --- | --- | --- | --- | --- | --- |
| Konaje stream |  |  |  |  |  |  |  |  |  |
| *Lunulospora curvula* Ingold | 30.1 | 15.8 | 32.5 | 38.3 | 31.8 | − | 57.8 | 34.1 | 30.8 |
| *Triscelophorus monosporus* Ingold | 38.4 | 33.3 | 38.8 | 40.0 | 16.7 | − | 14.7 | 25.6 | − |
| *Flagellospora curvula* Ingold | 17.8 | 33.3 | 7.5 | 3.3 | 25.8 | 4.3 | − | 15.9 | 42.3 |
| *Anguillospora longissima* (Sacc. & P. Syd.) Ingold | 5.5 | 15.8 | 11.3 | 13.3 | 15.2 | 8.5 | 10.8 | 3.7 | 23.1 |
| *Phalangispora constricta* Nawaei & J. Webster | − | − | − | − | − | 38.3 | − | − | − |
| *Trifurcospora irregularis* (Matsush.) K. Ando & Tubaki | − | − | 2.5 | 3.3 | 1.5 | 25.5 | − | − | − |
| *Flagellospora penicillioides* Ingold | − | 1.8 | − | − | 1.5 | 6.4 | − | 8.5 | − |
| *Anguillospora crassa* Ingold | − | − | 3.8 | − | 6.1 | 2.1 | − | 11.0 | 3.8 |
| *Triscelophorus konajensis*  K.R. Sridhar & Kaver. | 8.2 | − | − | − | − | − | 9.8 | − | − |
| *Ypsilina graminea* (Ingold, P.J. McDougall & Dann)  Descals, J. Webster & Marvanová | − | − | 2.5 | − | − | 5.3 | − | − | − |
| *Triscelophorus acuminatus* Nawawi | − | − | − | − | − | − | 5.9 | − | − |
| *Condylospora spumigena* Nawawi | − | − | 1.3 | 1.7 | − | 3.2 | − | − | − |
| *Lemonniera terrestris* Tubaki | − | − | − | − | − | 4.3 | − | − | − |
| *Clavariopsis aquatica* De Wild. | − | − | − | − | 1.5 | 2.1 | 1.0 | − | − |
| *Clavariana aquatica* Nawawi | − | − | − | − | − | − | − | 1.2 | − |
| Sampaje stream |  |  |  |  |  |  |  |  |  |
| *Triscelophorus acuminatus* Nawawi | 20.6 | 44.3 | 25.0 | 1.1 | 16.0 | 9.3 | 28.3 | 25.0 | 32.0 |
| *Lunulospora cymbiformis* K. Miura | − | 11.4 | 18.2 | − | 27.4 | 21.3 | 26.0 | 35.5 | 28.0 |
| *Anguillospora longissima* (Sacc. & P. Syd.) Ingold | 7.9 | 21.4 | 5.7 | 12.5 | 5.1 | 15.3 | 11.8 | 13.2 | 10.0 |
| *Lunulospora curvula* Ingold | 15.9 | − | 38.6 | 9.1 | 1.1 | 6.6 | 3.1 | 11.8 | 8.0 |
| *Flagellospora curvula* Ingold | 11.1 | 1.4 | − | − | 1.1 | 3.8 | 5.5 | − | 10.0 |
| *Brachiosphaera tropicalis* Nawawi | − | − | − | 1.1 | 7.4 | 3.3 | − | − | − |
| *Campylospora chaetocladia* Ranzoni | 1.6 | − | 1.1 | 1.1 | 5.1 | 6.6 | 2.4 | 1.3 | − |
| *Triscelophorus monosporus* Ingold | − | − | − | − | 2.9 | 2.7 | 7.9 | 1.3 | 6.0 |
| *Cylindrocarpon* sp. | 1.6 | 12.9 | − | 2.3 | 5.1 | − | 3.1 | − | − |
| *Helicosporium* sp. | − | − | 1.1 | − | 4.0 | 3.3 | 1.6 | 6.6 | 2.0 |
| *Clavariopsis aquatica* De Wild. | 9.5 | 4.3 | − | − | 1.1 | 2.7 | 3.1 | 1.3 | − |
| *Magdalaenaea Monogramma* G. Arnaud | 31.7 | − | − | − | − | 0.5 | − | − | − |
| *Flabellospora verticillata* Alas. | − | − | − | 5.7 | 2.3 | 4.4 | 0.8 | − | − |
| *Dactylella submersa* (Ingold). Sv. Nilsson | − | − | − | − | 2.9 | 3.3 | − | − | − |
| *Flabellospora crassa* Alas. | − | − | 1.1 | 1.1 | 2.9 | 1.1 | − | − | − |
| *Lemonniera terrestris* Tubaki | − | − | 8.0 | 2.3 | 1.1 | − | − | 1.3 | − |
| *Phalangispora bharathensis* T.S.K. Prasad & Bhat | − | − | 1.1 | − | 1.7 | 1.1 | 3.1 | − | 2.0 |
| *Isthmotricladia gombakiensis* Nawawi | − | − | − | − | 1.1 | 2.7 | − | 1.3 | − |
| *Triscelophorus konajensis* K.R. Sridhar & Kaver. | − | 1.4 | − | 2.3 | − | 2.2 | 2.4 | − | 2.0 |
| *Condylospora spumigena* Nawawi | − | − | − | 2.3 | 1.7 | 1.6 | − | − | − |
| *Trifurcospora irregularis* (Matsush.) K. Ando & Tubaki | − | − | − | − | 1.1 | 1.6 | 0.8 | − | − |
| *Clavatospora tentacula* Sv. Nilsson | − | − | − | 1.1 | 1.7 | 1.1 | − | − | − |
| *Phalangispora constricta* Nawawi & J. Webster | − | − | − | − | 1.1 | 1.6 | − | − | − |
| *Anguillospora crassa* Ingold | − | 1.4 | − | 14.8 | − | 1.6 | − | − | − |
| *Dwayaangam* sp. | − | − | − | 5.7 | − | 1.6 | − | − | − |
| *Flabellospora multiradiata* Nawawi | − | − | − | 4.5 | − | 0.5 | − | − | − |
| *Campylospora parvula* Kuzuha | − | − | − | 1.1 | − | − | − | − | − |
| *Campylospora* sp. | − | − | − | − | 0.6 | − | − | − | − |
| *Isthmotricladia laeensis* Matsush. | − | − | − | 2.3 | − | − | − | 1.3 | − |
| *Trinacrium* sp. | − | 1.4 | − | − | − | − | − | − | − |
